# “Why would you want to stand?” an account of the lived experience of employees taking part in a workplace sit-stand desk intervention

**DOI:** 10.1186/s12889-019-8038-9

**Published:** 2019-12-17

**Authors:** Jennifer Hall, Tess Kay, Alison McConnell, Louise Mansfield

**Affiliations:** 1 Department of Life Sciences, Brunel University London, Kingston Lane, Uxbridge, Middlesex UK; 2 Bradford Institute for Health Research, Bradford Teaching Hospitals NHS Foundation Trust, Duckworth Lane, Bradford, UK; 3 Faculty of Health Sciences and Sport, University of Stirling, Stirling, UK; 4 The Burrow, Auckland Road, Christchurch, BH23 4HH UK

**Keywords:** UK, Qualitative, Sedentary behaviour, Sitting, Standing, Physical activity, Multi-component intervention, Workplace health, Organisational culture, Product design

## Abstract

**Background:**

Sit-stand desk interventions have the potential to reduce workplace sedentary behaviour and improve employee health. However, the extent of sit-stand desk use varies between employees and in different organisational contexts. Framed by organisational cultural theory and product design theory, this study examined employees’ lived experience of taking part in a workplace sit-stand desk intervention, to understand the processes influencing feasibility and acceptability.

**Methods:**

Participant observations and qualitative interviews were conducted with 15 employees from two office-based workplaces in the UK, as part of a process evaluation that ran alongside a pilot RCT of a workplace sit-stand desk intervention. Observational field notes and transcripts were analysed using thematic analysis.

**Results:**

Three themes related to the experience of using a sit-stand desk at work were generated: employees’ relationship with their sit-stand desk; aspirations and outcomes related to employee health and productivity; and cultural norms and interpersonal relationships. The perceived usability of the desk varied depending on how employees interacted with the desk within their personal and organisational context. Employees reported that the perceived influence of the desk on their productivity levels shaped use of the desk; those who perceived that standing increased energy and alertness tended to stand more often. Sit-stand desks were voiced as being more acceptable than intervention strategies that involve leaving the desk, as productivity was conflated with being at the desk.

**Conclusions:**

The findings indicate a range of organisational, social-cultural and individual-level factors that shape the feasibility and acceptability of sit-stand desk use, and suggest strategies for improving employees’ experiences of using a sit-stand desk at work, which might positively influence sedentary behaviour reduction and health.

**Trial registration:**

Clinicaltrials.gov identifier NCT02172599, 22nd June 2014 (prospectively registered).

## Background

Being physically active is associated with reduced risk of premature all-cause mortality [1] and reduced risk of developing various health conditions including heart disease, diabetes, some cancers, and depression [2, 3]. Conversely, sedentary behaviour, which refers to sitting or lying behaviour whilst also not being otherwise physically active (energy expenditure < 1.5 METs), is associated with *increased* risk of premature all-cause mortality and poor health [4, 5]. Office-workers spend a greater proportion of work hours sat down compared to non-work hours (68% vs 60%) [6] given the desk-based nature of office work. Thus, office-based workplaces are an important setting for intervention to offset the negative health consequences of inactivity and prolonged sedentary behaviour. The current UK governmental guidelines for physical activity recommend that adults “minimise the amount of time spent being sedentary (sitting) for extended periods” [7] and a recent expert consensus statement regarding workplace sitting specifically recommends that office-workers should incorporate at least 2 h/day of standing or light activity into work hours, progressing to 4 h/day [8].

The provision of sit-stand desks, i.e. height-adjustable desks that the user can sit *and* stand at, is an efficacious strategy to reduce and break up office-based sitting. A recent systematic review of workplace interventions reported a significant reduction in sitting in *all* studies involving environmental change (*n* = 6; sitting reduction ranged from 28.8 to 104.1 min/work-day), compared to only 20% of studies that were focused on educational or behavioural strategies (*n* = 15) [9]. Five of the six studies reported sit-stand desk provision as the environmental change strategy [9]. However, the extent of sit-stand desk use varies in different organisational and workplace contexts [10, 11]. The socioecological model comprehensively integrates the individual and the social to represent a framework that recognises a multitude of interconnected factors on different ‘levels’ (e.g. individual, organisational, societal) that influence attitudes, values and behaviours [12]. In other words, workplace initiatives aimed at reducing sitting are not isolated events but sit within the wider practices of the organisation and employees’ lives [13]. Utilising of the socioecological model thus permits an investigation of how the intervention context interacts with sit-stand desk provision to influence sit-stand desk use. Examining the processes influencing the feasibility and acceptability of sit-stand desk use through accounts of employees’ lived experiences in different organisational contexts, will aid understanding of the appropriateness of sit-stand desks as a workplace health strategy [14].

Several qualitative studies have examined employees’ experiences of using sit-stand desks (e.g. [15–18]). A recent review and thematic synthesis identified factors influencing the feasibility and acceptability of reducing occupational sitting across all domains of the socio-ecological model [19]. Interweaving theoretical reasoning with the empirical data can add explanatory value as to the processes that underpin employees’ narratives and actions [20]. However, previous investigations are mostly descriptive of employees’ experiences and tend to emphasise factors influencing behaviour at the level of the individual (e.g. [15, 16]). Organisational cultural theory is a framework for understanding how people think, feel and act within the workplace context [21]. Organisational culture is functional in that it offers an “interpretation of an institution’s history that members can use to decipher how they will be expected to behave” ([22], p., 52); it represents a regulatory mechanism concerning employee workplace conduct. Utilising an organisational cultural theoretical lens situates employees’ experiences and perceptions within the web of social, organisational and societal influences on behaviour, which aligns with the socioecological model. While the socio-ecological model provides a structure for analysis, organisational cultural theory can be applied to help *explain* how workplace cultural dynamics facilitate or restrict sit-stand desk use, and how the provision of sit-stand desks can alter workplace (sitting) practices through the disruption of cultural dynamics [13].

Additionally, whilst sit-stand desks are a commercially available product, existing research relating to sit-stand desk use has lacked engagement with product design theory. Products do not control behaviour, but rather how a person interacts with a product in a specific context influences lived experience, and products can transform behaviours [23, 24]. A user’s emotional response to a user-product interaction is one of the most significant contributors to overall product experience and (dis) continuation of product use [25]. The application of product design theory and literature to empirical investigations of lived experiences of sit-stand desk use may elicit a more comprehensive understanding of the feasibility and acceptability of sit-stand desks as a workplace initiative. The aim of the present study was to examine the processes that influence the feasibility and acceptability of sit-stand desk use, using qualitative interview and observation methods and framed by organisational cultural and product design theory and the socio-ecological model, amongst office-based employees in two UK non-profit organisations.

## Methods

### Background to the study

The work reported herein forms part of a wider programme of work that involved the delivery and evaluation of a multi-component sit-stand desk intervention via a pilot randomised controlled trial (RCT), and a process evaluation, within two participating organisations. Both organisations are non-profit and have open-plan office spaces over multiple floors accommodating around 1000 employees. Workplace A is a health charity and Workplace B is a national health-related Governmental organisation.

#### Multi-component intervention (SS-MC)

**Fig. 1:**
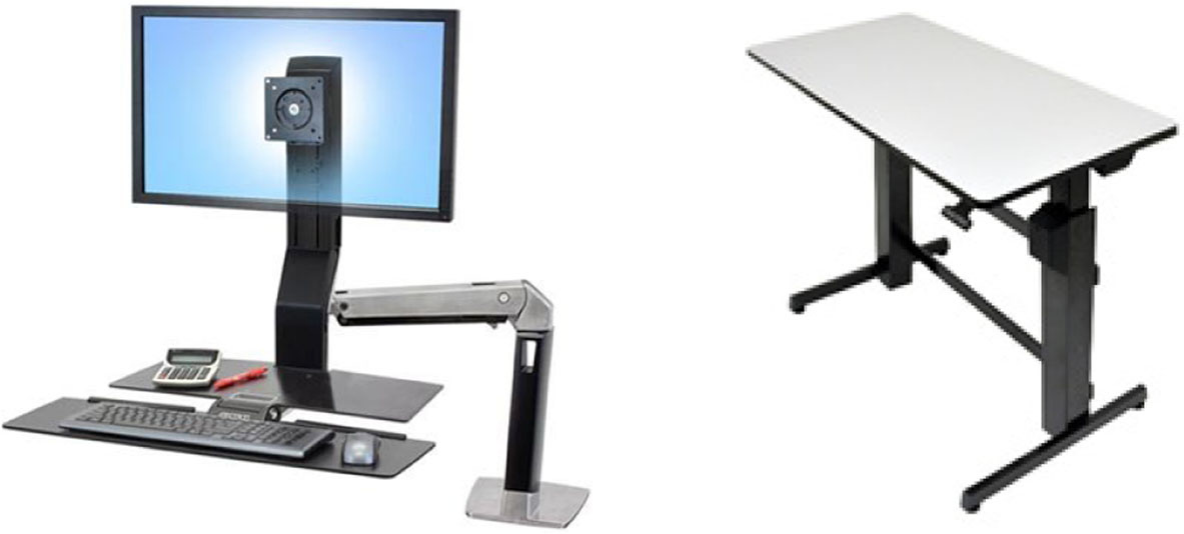
Depiction of Ergotron Workfit-A (left) and Ergotron Workfit-D (right). Published with permission from www.ergotron.com

The intervention was devised based on the socio-ecological model; targeting multiple levels of influence is more likely to result in a change in behaviour than targeting one level of influence alone [26]. The 6-month intervention included organisational, environmental, and individual-level strategies. The organisational-level strategy consisted of a series of four emails sent from organisational managers, including content relating to the organisations’ commitment to creating a healthy working environment. The environmental-level strategy involved the provision of a sit-stand desk. Participants were given a choice between two models; an Ergotron workfit-A (sit-stand workstation) or an Ergotron workfit-D (sit-stand desk); see Fig. 1. Participants were given the opportunity to try each model prior to making their choice. The individual-level procedures included the delivery of four brief motivational-interview based phone calls designed to support participants to overcome barriers to using the sit-stand desk. The intervention incorporated 12 behaviour change techniques (BCTs) [27]; see Table 1.

**Table 1:** An overview of the content and BCTs employed within the multicomponent intervention.* BCTs as described in Michie, Ashford, et al. [27]

Strategy	Level	Behaviour Change Strategies / Content
Phone call 1 (baseline - time 0)	Individual	Motivational Interviewing* – phone calls conducted according to motivational interviewing principles of engaging, guiding and evoking Providing information of consequences of physical activity and sedentary behaviour* – discussed the health risks of sedentary behaviour and the benefits of physical activity Barrier identification* – participants were asked if they could anticipate any barriers with using their sit-stand workstation. Challenges were discussed and minimised where possible
Management email 1 (2 weeks)	Organisational	*Content*: organisation wish to create a ‘healthy’ working environment, sit-stand workstations potentially create a healthier working environment
Sit-stand workstation installation, ergonomic briefing (3 weeks)	Environmental	Environmental restructuring* – usual seated desks converted into, or replaced by sit-stand desks (Ergotron workfit-A or workfit-D) Model / demonstrate the behaviour* – A researcher physically demonstrated how to use the sit-stand desk Provide instruction on how to perform the behaviour* – A researcher verbally provided instructions on how to use the sit-stand desk
Phone call 2 (5 weeks)	Individual	Motivational Interviewing* - phone calls conducted according to motivational interviewing principles of engaging, guiding and evoking Barrier identification* - participants were asked if they experienced any barriers with using their sit-stand workstation. Challenges were discussed and minimised where possible Provide instruction on how to perform the behaviour* – Participants were given tips on ‘how to stand’ including: regular switching between sitting and standing, taking breaks from the computer, wearing comfortable footwear, and correct posture Goal setting (outcome or behaviour)* – Participants were given the opportunity to set goals of their choice (e.g. reducing sitting by 2 h a day)
Management email 2 (8 weeks)	Organisational	*Content*: physical and psychological health of employees is a priority for the organisation, a poster providing information on how using sit-stand workstation (and reducing sedentary behaviour and increasing physical activity) could benefit health at work
Phone call 3 (12 weeks)	Individual	Motivational interviewing* - phone calls conducted according to motivational interviewing principles of engaging, guiding and evoking Prompt review of (behavioural or outcome) goals* – Where set, participants were asked whether they had met their goals Prompt self-monitoring of behaviour / Prompt practice* – participants were advised to prompt and monitor their behaviour using a method of their choice
Management email 3 (16 weeks)	Organisational	*Content*: development of healthy work environment can lead to healthier workforce and organisational improvements including improved productivity and enhanced outcomes, the philosophy of the organisation is aligned with Governmental workplace health policies
Phone call 4 (21 weeks)	Individual	Motivational interviewing* - phone calls conducted according to motivational interviewing principles of engaging, guiding and evoking Relapse prevention* – A discussion of how participants can avoid ‘breaking the habit’ of using their sit-stand desk, especially after spending prolonged time away from the workplace / desk Prompt generalisation of a target behaviour* – A discussion of how to incorporate less sedentary behaviour and more physical activity into other areas of life and work activities
Management email 4 (6 months)	Organisational	*Content*: societal shift towards reducing prolonged sitting in the workplace, organisation as a pioneer of this societal shift, encouragement for participants to advocate the approach

#### Pilot RCT

The pilot RCT employed two intervention arms: (1) SS-MC described above; and (2) sit-stand desk only (SS-O). Participants in the sit-stand desk only arm received their choice of sit-stand desk but did not receive any other elements of the multi-component intervention. A control arm for usual desk-based working practice (no sit-stand desk) was also included. Thirty employees were recruited and randomised into one of three study arms (*n* = 10 per arm). Randomisation was performed separately for each site to ensure an equal proportion of participants in each arm, per organisation.

#### Process evaluation

The process evaluation – underpinned by the MRC Guidelines for evaluating complex interventions [28] – set out to examine (1) the feasibility and acceptability of sit-stand desk implementation from the perspective of organisational stakeholders, and (2) the feasibility and acceptability of sit-stand desk use from the perspective of intervention participants. The process evaluation comprised qualitative methods, to permit an examination of processes underpinning how the intervention was experienced based on the interaction between the intervention and the delivery context [29]. Participant observation was conducted to examine organisational cultural and contextual factors underpinning feasibility and acceptability. Ethnographic principles, including immersion and participation within the organisational setting, taking a collaborative approach, and utilising introspection and reflexivity [30], guided the observational data collection. Within this project the lead author undertook a volunteer role within the two workplaces that the intervention was implemented, to engage in participant observation and be a ‘participant’ (employee) within the workplace. Engaging in behaviours appropriate to the setting, rather than simply observing, can facilitate a more nuanced understanding of meanings attached to behaviours ([31] p. 61). Qualitative semi-structured interviews were also undertaken by the lead author with research participants from the participating organisations to understand their experiences. Herein, this paper reports the methods and findings related to the second aim of the process evaluation: the feasibility and acceptability of sit-stand desk use from the perspective of intervention participants. The organisational stakeholder findings are described in a separate paper [32].

### Recruitment and sampling

Employee interviewees were a subsample of participants that took part in the pilot RCT of the workplace sit-stand desk intervention. Inclusion criteria for participation in the pilot RCT included: no use of a sit-stand desk in the last 4 weeks, being capable of standing, being full-time employed on a permanent or fixed term contract until the anticipated study end date, with no plans to leave the organisation, or be absent for an extended period (≥ 4 weeks), being present at the worksite ≥4 days a week, and being at least 18 years of age. Participants were recruited via internal advertisement (email, yammer (a social network platform for workplaces), posters). Those that expressed interest were invited to a recruitment workshop (60 min) at their organisation and subsequently complete an expression of interest form. Sixty-eight employees attended a workshop and 43 completed an expression of interest form. Based on a pre-specified sample size for the pilot RCT, 30 of these employees were recruited and provided written informed consent.

Of these 30 employees, 17 were invited to take part in an interview (*n* = 2 declined). Interviewees were purposely selected to ensure representation of a diversity of views, according to gender, age, ethnicity, job role and seniority, intervention arm, organisation, and experience of using the sit-stand desk: including frequency / duration of use in the standing position, and whether the experience was positive or negative. Knowledge of participants’ experience of using the sit-stand desk was attained through systematic participant observation, and ongoing informal conversations with participants. We judged this interview sample to be appropriate to generate high quality in-depth data, of sufficient quantity, to address the research question. An overview of the characteristics of the participant interviewees can be found in Table 2.

**Table 2:** Summary of participant characteristics

	Workplace A number (%)	Workplace B number (%)	Total number (%)
Study group			
SS-MC	2 (25.0)	3 (42.9)	5 (33.3)
SS-O	4 (50.0)	3 (42.9)	7 (46.7)
Control	2 (25.0)	1 (14.3)	3 (20.0)
Gender			
Female	6 (75.0)	4 (57.1)	10 (66.7)
Male	2 (25.0)	3 (42.9)	5 (33.3)
Age			
16–24	1 (12.5)	0 (0.0)	1 (6.7)
25–34	3 (37.5)	2 (28.6)	5 (33.3)
35–44	2 (25.0)	2 (28.6)	4 (26.7)
45–54	2 (25.0)	1 (14.3)	3 (20.0)
55–64	0 (0.0)	2 (28.6)	2 (13.3)
Ethnicity			
White	7 (87.5)	5 (71.4)	12 (80.0)
Asian/Asian British	0 (0.0)	1 (14.3)	1 (6.7)
Black/Black British/African/Caribbean	1 (12.5)	0 (0.0)	1 (6.7)
Other ethnic group	0 (0.0)	1 (14.3)	1 (6.7)
Personal monthly income (before tax)			
£870–£1500	1 (12.5)	0 (0.0)	1 (6.7)
£1500–£2400	3 (37.5)	1 (14.3)	4 (26.7)
£2400–£3900	3 (37.5)	4 (57.1)	7 (46.7)
£3900+	1 (12.5)	2 (28.6)	3 (20.0)
Sexual orientation			
Heterosexual	6 (75.0)	4 (57.1)	10 (66.7)
Gay / Lesbian	0 (0.0)	3 (42.9)	3 (20.0)
Bisexual	1 (12.5)	0 (0.0)	1 (6.7)
Other	1 (12.5)	0 (0.0)	1 (6.7)

The job roles of participants have not been included in Table 2 to preserve their anonymity, however their roles ranged from project assistants and officers, to programme leads (middle management) to heads of directorates (senior management).

### Data collection

Observations involved three formal phases, each consisting of 9–13 ‘working’ days in each workplace. Additionally, informal participant observations and interactions have occurred, been recorded and utilised in the analysis throughout the duration of involvement with both organisations. The observations focused upon activities, employees’ behaviours and interactions, and the workplace setting [31]. One hundred forty-seven thousand six hundred sixteen words of field notes based on the formal observation phases, and 37, 750 words of field notes based on informal observations and were recorded.

The interview guides were both theoretically and empirically informed; they were shaped by the observational data that had been collected during initial observations. Two pilot participant interviews were conducted with employees at an organisation that *wasn’t* participating in the intervention study; one that has a sit-stand desk and another that has a seated desk within an open-plan office. The interview guides were modified following the pilot interviews to include the addition of questions regarding sit-stand desks and productivity, as the pilot interview data suggested that views related to productivity and organisational culture might be linked to the acceptability of sit-stand desk use. See Additional file 1 for example interview guides for intervention group and control group participant interviewees. The interviews were mainly conducted face to face within a meeting room in the interviewee’s workplace or at a nearby café. However, three interviews were conducted over the telephone, upon the request of the interviewee. Interviews took place with 15 participants approximately 7 months following the installation of sit-stand desks within their organisation. The interviews were 38 min long on average, ranging from 20 to 57 min.

### Data analysis

A reflexive thematic analysis was utilised to collectively analyse the interview and observational data. Approximately half of the interviews were transcribed by the lead author to facilitate data immersion [33], with the remaining interviews being transcribed by a professional transcription company. NVivo 10.0 software was utilised to manage the data analysis; the analysis process undertaken aligned with the steps outlined by Braun and Clarke: data familiarisation, generating initial codes, searching for themes, reviewing, refining and defining themes [33]. The data were analysed by synthesising theory and evidence and thus involved both induction (i.e. being data-driven) and deduction (directed by existing theory, concepts, ideas). During the initial coding there was a primary focus on the data, which was then related to theoretical constructs, as this type of theory-data interplay permits new knowledge and insights to be generated [34]. Thus, the data were coded openly, with the socio-ecological model, organisational cultural theory, and product design theory being utilised as ‘sensitising concepts’, rather than data being coded into pre-defined theoretically-informed codes or themes. The lead author (JH) reviewed and coded all raw data and another author (LM) independently double-coded 20% of the raw data; the authors met to compare codes and discuss differing interpretations, to facilitate a richer analysis [35]. The lead author (JH) crafted themes from the coded data which related to employees’ experiences of sit-stand desk use. The themes were reviewed, refined and agreed by all researchers (JH, LM, TK, AM). The themes were further refined based on peer-review feedback. These themes are presented as findings. Pseudonyms are used within data extracts provided to support the analysis to maintain participant anonymity.

## Results

Six themes related to processes influencing the feasibility and acceptability of sit-stand desk use include: (1) behaviour change and habit formation, (2) sit-stand desk design, (3) employee health: expectations and outcomes, (4) the prioritisation of productivity, (5) social norms and interpersonal relationships, and (6) normalisation of standing: organisational cultural change. These themes, whilst overlapping, are described in turn.

### Behaviour change and habit formation

Interviewees’ accounts indicate that sit-stand desks can contribute to behavioural change by challenging the habituated and routinely performed practice of sitting at the desk to work. Steph explained “*it’s so obvious that it is a different desk … you know you’re sitting at it and you sort of look down and go ‘oh yeah, I could stand up’ …*” (Steph, Workplace A, SS-O). Melissa described *“[initially], it was a bit of a novelty, you know … I’d stand for like three, four hours”* (Melissa, Workplace B, SS-MC). After the initial novelty effect diminished, employees reported a need to make a conscious effort to develop a practice of standing at the desk. Employees described different strategies that they adopted to encourage regular postural change at the desk, including task-based (choosing to stand for some tasks and sit for others, or changing posture following the completion of a given task), bodily cues (switching position upon experiencing physical discomfort or mental tiredness) or time-based (changing posture after a given length of time, or standing at certain times of day):

If I've got a report or if I've got a big chunk of emails … In my diary I would, I wouldn't put sit-stand, but for me I knew … . For me it seems to be more around the types of work. That seems to be sticking more for me (Melissa, Workplace B, SS-MC)

I would do an hour or two in the morning, and then sit down, and then again in the afternoon before I go home I would do an hour or two hours (Brett, Workplace B, SS-MC)

Some participants reported that standing at the desk became habitual, relying less on deliberate strategies of use over time. However, others still had to exert conscious effort after 7–8 months of being given the desk:

So, it's still, I still haven't quite got into a pattern [of using the sit-stand desk]. I'm still consciously I suppose, thinking during the day. And I was thinking even this morning (Melissa, Workplace B, SS-MC)

### Sit-stand desk design

Design features, which varied between sit-stand desk models, influenced participants’ experience and use of the desks. Whilst some employees thought the sit-stand desks were aesthetically appealing, others were dissatisfied with their appearance. Cristina commented that the workfit-D *“just felt really old fashioned … clunky and just quite plasticky, metally … cheap”* (Cristina, Workplace A, SS-MC). Steph commented, *“the second one [workfit-A] looks like a bit of a crazy transformer as well, I don’t know if it’s a bit more medical looking, yeah... just cos it’s a big kind of robotic thing whereas the other one [workfit-D] was basically just a desk*” (Steph, Workplace A, SS-O). The look and feel of the desks elicited emotional reactions amongst employees, which influenced their attitudes towards using them. For example, Steph assigned the two desks opposing personalities; *“The first one [workfit-D] is friendly and the second one [workfit-A] is a bit more like, mean”* (Steph, Workplace A, SS-O). Employees preferred a more standard looking desk, which led to the evaluation of the workfit-A as more aesthetically harmful than the workfit-D.

Aspects related to the sit-stand desk design including the size of the desk, instability of the keyboard tray (workfit-D), and the size and instability of the work surface (workfit-A), impeded use of the sit-stand desks for completing work efficiently:

I really, really hate the size of the desk [workfit-D], it could just be because I'm used to having a really big desk but … you can only have like two bits of paper and your phone and I think its significantly smaller than the other one [original seated desk]. And also … the key board is placed, it’s really low, so I have to put the keyboard on the desk (Anita, Workplace A, SS-O)

The only thing was that it [workfit-A] bounced quite a bit … not quite feeling like a firm table. That was another one of the reasons why I thought maybe the table [workfit-D] would have been better (Sean, Workplace B, SS-O)

Users’ experiences of the sit-stand desk were affected by their own practices. For example, employees who put more pressure on the keyboard when typing reported that the workfit-A surface felt unstable, which reduced the ease of use:

The biggest issue (in Craig’s opinion) is that the arm moves down whilst people are typing! He says that the workforce at Workplace B is quite ‘old’ and they ‘don’t have many people that did GCSE ICT’ and so they ‘prod’ the keys heavily, which is probably putting quite a lot of weight on it, and exacerbates the issue of instability (Research notes, Workplace B, 5^th^ Sept. 2015).

Employees that expressed a preference or requirement for working with hard copies of documents - rather than electronic versions - were more likely to identify the size of the desk as an issue. Conversely, Workplace B employees were less likely to note the desk size as a limitation due to the enforcement of an organisation-wide clear-desk policy:

[I thought] Oh my God, that’s so small *laughs* … when I realised how small it was, I was a bit freaked out … like I’ve got so many papers, and different things so I need more space … it’s the way I prefer to work (Carol, Workplace A, SS-O)

I have a bit less space on the desk … I think that's a benefit … you can't hoard things … I am conscious of having a cleaner desk (Melissa, Workplace B, SS-MC)

### Employee health: expectations and outcomes

Most employees that volunteered to participate in the sit-stand desk trial expressed a desire to use a sit-stand desk to reduce prolonged sitting to prevent or manage occupational – primarily musculoskeletal - health issues. Some employees had experienced discomfort in the lower body that they attributed to sitting at the desk, whereas others were wary of the *potential* risks associated with prolonged workplace sitting:

My mums got a bad back and other members of my family … I'm going to be susceptible to it, and then not make it any better myself through the way that I sit … [so] I thought maybe actually by standing up, I would get some health benefits out of that (Grace, Workplace A, control group)

In practice, using a sit-stand desk led to a variety of physical responses, including increased and decreased musculoskeletal discomfort (MSD). Employees that experienced MSD because of, or exacerbated by, prolonged workplace sitting tended to report that using the sit-stand desk reduced discomfort. For example, Nadia commented *“when I’m standing it’s made a big difference [reduced back pain]. It’s really, really improved”* (Nadia, Workplace B, SS-O). Conversely, those that did not report pre-existing occupational health issues were more likely to experience MSD when standing:

After long periods [of standing] ... I mean it sort of goes all the way up your back, this bit of strain … and not in a good sense, like when you've been doing lots of physical activity and it's a good strain and a good aching … that was uncomfortable, I found … I thought maybe standing up would be, would be better (Sean, Workplace B, SS-O)

Some employees did not act upon the regular postural switching advice given during a sit-stand desk ergonomic assessment, which led to the occurrence of MSD and negatively influenced some employees’ experience.

Despite physical health concerns being the primary driver, some employees also referred to the potential *physiological* health risks of high levels of, and uninterrupted, sitting as a reason for wanting to use a sit-stand desk to reduce sitting:

Having had that sort of, um, knowledge about it, the actual impact of sitting for several hours, being inactive... All that that potentially does to the inflammatory response system that then sort of made me think I need to be standing (Nadia, Workplace B, SS-O)

Caroline commented that she was interested in the concept of sit-stand desks, as she *“had become aware that sitting all day isn’t particularly good for you, I’ve read stuff in the media about the negative effects on your health”* (Caroline, Workplace A, SS-O).

Employees that valued (potential or experienced) health *benefits* of using a sit-stand desk were less likely to succumb to barriers to using the sit-stand desk to stand, and more likely to adapt the way they work to accommodate the sit-stand desk:

She told me that she wore her winter boots yesterday and that they have a slight heel, and that she found that she couldn’t really stand for long with them on as it wasn’t very comfortable. So, she said that she would not wear them anymore … as she wants to stand more and takes her own wellbeing incredibly seriously … . she will sacrifice things to be able to do that (Research notes, Workplace B, 4^th^ Nov. 2015)

I’ve had to adapt the way that I work [to the smaller desk size], so I won't spread things out … [or] if there’s a quiet room I've gone and done that there (Nadia, Workplace B, SS-O)

### The prioritisation of productivity

Employees were compelled by a normative power to limit breaks from the desk for fear of being perceived as unproductive by their colleagues:

When you're managing your own time... when you've got work that needs to be done … it [taking a lunch break] can look like, ‘we've got loads to do, why're you going off for an hour’?... or if you said, ‘I'm really stressed, I'm really busy’ and then went off for 10 minutes, they would be like, ‘what are you doing’ (Grace, Workplace A, control group)

The data illustrates that office-based employees conflate productivity with sitting at the desk. Hence, valuing productivity perpetuates cultural beliefs and behaviours related to workplace sitting; taking breaks is symbolised as time-wasting. The amalgamation of work prioritisation values with worker autonomy (i.e. employees being responsible for managing their own time) can invoke employee behaviours such as working long hours without breaks. The perception that doing and being at work centres around sitting at the desk led employees to express positive attitudes towards sit-stand desks, compared to other potential strategies to reduce workplace sitting:

Having the [sit-stand] desk would allow me to, to change positions and still do my work and not take distraction away from work (Reece, Workplace B, control group)

Sit-stand desk provision is perceived to be a suitable strategy to reduce workplace sitting as sit-stand desk use does not compromise work activity or necessitate leaving the desk. However, employees varied in whether they felt the sit-stand desk allowed them to be more, equally, or less productive in work. For example, some employees found they were better able to concentrate when standing compared to sitting, and reported that standing increased their energy and alertness:

If I'm feeling a bit fatigued, once I stand I feel more alert. So, if I've got written work to finish, that [standing] really does help me and I think it does give me that impetus [to complete work] (Melissa, Workplace B, SS-MC)

Other employees, however, reported that they found standing more distracting than sitting, which had a negative impact on their work efficiency:

I'm defaulting to sitting down more now. Um, I think that's mainly because of the, certain pieces of work I’ve been doing require concentration … it's easier to concentrate sitting down (Sean, Workplace B, SS-O)

The impact of using a sit-stand desk on work productivity was a particularly salient issue as employees universally described an organisational norm of intense work and maximising productivity. Workplace A employees’ desire for efficiency was driven by an internal motivation to support people affected by cancer, whereas Workplace B employees’ high workload stemmed from governmental cost-saving measures:

We're still in the process of going through a restructure and that kind of stuff … some of us have done the job of two or three people so you're so focused on coming to work, doing your work, doing as much as you can and go home (Brett, Workplace B, SS-MC)

Given the strong organisational norm of maximising productivity, the influence of using the sit-stand desk on work efficiency was strongly associated with sit-stand desk use; employees chose to stand more if they felt standing has a positive impact on productivity, whereas a (perceived or actual) reduction in productivity reduced the acceptability of standing at work.

### Social norms and interpersonal relationships

Cultural norms regarding workplace, and particularly desk-based sitting, negatively influenced some employees’ sit-stand desk experience. Specifically, workplace sitting norms incited the shared symbolisation of standing at the desk as abnormal by employees:

Genuinely people are … ‘why would you want to stand?’ … people just think it’s a bit strange (Mark, Workplace A, SS-MC)

Being perceived as ‘strange’ by colleagues engendered social discomfort and feelings of self-consciousness for some sit-stand desk users, which reduced their acceptance of standing and compelled them to conform to the shared norm of sitting at the desk. Some sit-stand desk users felt that standing in the workplace gave the impression of looking down on colleagues, which made them feel uneasy:

Someone remarked that it [standing at the sit-stand desk] was a bit like a headmistress looking over everyone (Research notes, Workplace B, 23^rd^ April 2015)

Nevertheless, standing at the desk can facilitate conversation between the sit-stand desk user and other employees by permitting interaction on one level:

If somebody comes to speak to me and I'm sat down, I stand up because otherwise it diminishes my power … its body language, you know... you can converse much more at your peer level than if you’re sat down... if you're already stood you're already inviting that person to communicate with you at that level and you get on (Bridget, Workplace B, SS-MC)

Walking and speaking to colleagues that are standing at their sit-stand desk may be more amenable than standing and speaking to colleagues that are sat at their desk, due to the implied power differentials between the two postures.

Some participant employees were wary of potential disruption to colleagues caused by them standing or transitioning between postures:

I was trying not to cause too much distraction, up and down … [but] now my team are used to me up and down during the day and I can see that even the person next to me, she'll still be doing what she's doing on her screen (Melissa, Workplace B, SS-MC)

She [a colleague of a sit-stand desk user] said that she found it a little distracting – [sit-stand desk user] moving her desk up and down – and especially having to strain to talk to her as she sits next to her (Research notes, Workplace B, 23^rd^ June 2014)

The data shows that, whilst employees *feared* that using the sit-stand desk would be a cause of disruption to colleagues, which contributed to discomfort amongst sit-stand desk users, disruption rarely materialised upon use of the desk.

Sit-stand desk users also sensed a perception of privilege amongst colleagues. Interviewees identified that their colleagues thought they had been given a superior desk which was not accessible to other employees, which led some sit-stand desk users to experience feelings of guilt. For example, some interviewees reported feeling guilty for sitting when they knew their colleagues were envious of their opportunity to stand:

We have a couple other people [in our team] … they just got, not jealous but I think they just had like really envious eyes … . when I first got mine, that definitely was the first feeling, like why have you got a standing desk if you're not going to use it … . definitely I felt guilty when I wasn't using it properly (Anita, Workplace A, SS-O)

Colleagues’ perceptions of special treatment directly influenced sit-stand desk users’ emotions.

### Normalisation of standing: organisational culture change

The installation of sit-stand desks initiated a process of normalisation of standing within the organisations. The presence of the sit-stand desks prompted informal conversation amongst sit-stand desk users and other employees:

People wanted to know a lot more about it in terms of the experience of it … People in the office, random, well not random but even people I don't know from the office will come over and ask. It's been quite a talking point, and I’ve got a bit evangelical about it now. I think everybody, if the option was there would be interested to try it (Melissa, Workplace B, SS-MC)

In addition, sit-stand desks were used by other employees when the owner was absent from the office, and some sit-stand desk users directly influenced the standing habits of other employees:

I think that initially I actually had people asking me if they could use the desk, while I'm not in the office and that's fine, no problems … When I've come in the next day it's been adjusted, so I know someone's been using it whilst I've been away (Nadia, Workplace B, SS-O)

He sent a calendar invite for a recurring meeting to his team, and the location said ‘standing somewhere’. It’s interesting to see that Peter, as a manager, may be influencing his team’s sitting and standing habits … some participants are leading with it [standing at work] and introducing the idea to others that hadn’t experienced it before and planting the seed that it is something that they could do (Research notes, Workplace A, 23^rd^ Feb 2015)

Bottom-up cultural processes contributed to the normalisation of standing and increased receptivity towards using a sit-stand desk amongst a wider range of employees not directly participating in the sit-stand desk trial.

The installation of sit-stand desks also contributed to a positive attitudinal shift of senior employees that would be instrumental in more widespread provision of sit-stand desks:

[senior employee] said her brother said it [a sit-stand desk in his office] was strange and that he’s never heard of it before. [senior employee] said that she said ‘Oh no it’s actually normal, we’re doing some work on it at [Workplace A], it’s going to be the next big thing, it’s going to be the future’ (Research notes, Workplace A, 4^th^ November 2014)

He did say that the project has initiated a change in cultural attitude within [workplace B] towards sit-stand [desks], which is hard to capture, but very important (Research notes, workplace B, 11^th^ November 2015)

Observations indicate that the sit-stand desk project has been instrumental in strengthening an organisational commitment to developing more opportunities for employees to reduce sitting at work:

The sit-stand project is helping to push the wellbeing agenda within the organisation. One thing they are doing is 10-minute PA/stretching sessions at lunch times … They are also looking for wellbeing champions (Research notes, Workplace A, 19^th^ June 2015)

## Discussion

**Fig. 2:**
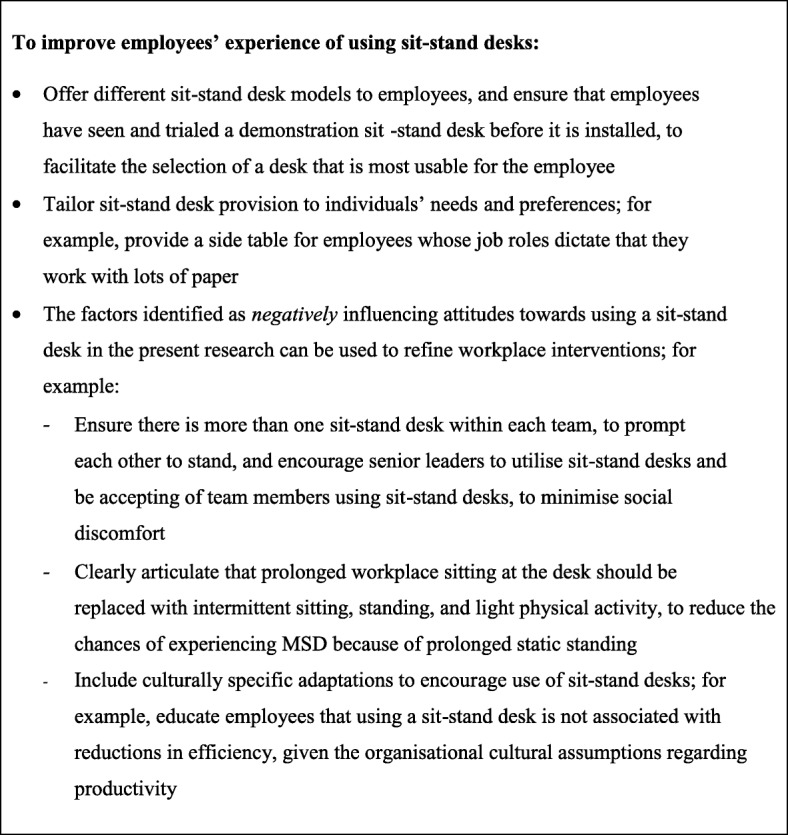
Providing sit-stand desks: Recommendations for workplaces

This study involved conducting observations and interviewing employees from two UK workplaces participating in a pilot RCT of a workplace sit-stand desk intervention. The aim of the study was to understand the web of cultural, organisational, social, environmental and individual-level factors influencing the feasibility and acceptability of using a sit-stand desk at work. Sit-stand desks contributed to behaviour change and habit formation amongst participants. The sit-stand desk design, the perceived influence of standing on health, organisational cultural assumptions relating to productivity, and workplace social dynamics were found to shape employees’ views related to their sit-stand desk. Sit-stand desk provision contributed to the normalisation of standing more broadly within the participating organisations. See Fig. 2 for a summary of recommendations for workplaces based on the findings reported in this paper.

For most office-based employees, seated desk-based working is a subconscious practice. It is theorised that practices are sustained by the reciprocal relationship between the objects, meanings and behaviours tied to practices [36]. The present study provides evidence that substituting a seated desk with a sit-stand desk (object) can weaken the link between sitting (behaviour) and doing productive work (meaning). In other words, the presence of a sit-stand desk can disrupt habituated workplace sitting by alerting attention to the opportunity to stand. However, the findings suggest the requirement for *conscious* habit-making and deliberate effort on behalf of the sit-stand desk user to initiate and *maintain* a standing practice at work. This contests the application of nudge theory to the influence of sit-stand desks on behaviour, which posits that users may alter their behaviour in response to the options provided by the product somewhat automatically, subverting a more complex decision-making process, and without conscious effort [37]. The present study identified strategies that sit-stand desk users adopt to facilitate a change in posture, including time-based, task-based, and comfort-based. Existing qualitative studies of sit-stand desk use have also identified these strategies as being key to facilitating use of the sit-stand desk in the standing position [16, 18]. The findings suggest that, in order to maximise sit-stand desk use and associated health benefits, workplaces should provide information to employees about different strategies for prompting postural change. An additional prompt reported in extant literature [18] – but not in the present study – is seeing others standing. It is plausible that there was limited opportunity for others’ standing to act as a prompt to stand in the present study as sit-stand desk users were relatively dispersed, including being located on different floors.

Design literature indicates that the perceived usability of a product, which incorporates efficiency, effectiveness and ease of use, strongly shapes employees’ attitudes towards the product [24]. Whilst the central purpose of sit-stand desks is reducing sitting and encouraging movement, it must also support employees to complete tasks by providing a flat and sufficiently large surface to place and utilise objects that are fundamental to the completion of those tasks, such as a computer and papers. Consistent with previous empirical research (e.g. [17, 18]) the present study indicates that design features including the sit-stand desk size and instability hinder the effectiveness of the desks for supporting completion of work, compared to employees’ original seated desk. People’s actions and experiences were influenced by the desk features (e.g. size, stability) organisational context (e.g. organisational policies, job-related tasks) and individuals’ health-related attitudes. These findings indicate that, in line with product design theory, there is a relationship between the user, product and context – and their interaction - which signifies product experience [38]. The findings suggest that there are minimum design requirements to enable workers to complete their work, however, beyond this employees engage their reflexivity to choose whether and how to respond to the sit-stand desk, whilst their choices are disposed by various structural factors that enable or impede use of the sit-stand desk [39]. The finding that some employees were willing to adapt how they work to accommodate the sit-stand desk, and some were not, is consistent with a recent qualitative study of current and past sit-stand desk users [40]. Organisations should consider models of sit-stand desks that suit their environment, and permit employees to trial and select models of sit-stand desk that best ‘fit’ their own needs and how they interact with their desk, to minimise the amount of adjustment required.

Some participants reported experiencing physical discomfort when standing statically at the desk for prolonged periods. Physical discomfort can be caused by standing for too long without having moving or sitting breaks [41]. Accordingly, an expert statement recommends that employees avoid prolonged, static standing postures [8]. However, there is a perception amongst the public that this recommendation is unclear and contradictory when positioned alongside guidance to use a sit-stand desk to reduce prolonged sitting [42]. Clear recommendations to utilise the sit-stand desks to regularly alternate between sitting and standing postures, and to incorporate movement, should be communicated to users.

In the present study, employees’ use of the sit-stand desk was contingent upon their perception of the influence it had on their productivity, given the organisational cultural value of maximising productivity. A recent systematic review of studies using quantitative measures of productivity concluded that there is no influence of sit-stand desk use on productivity [43]. Findings from the present study suggest that the relationship between sit-stand desk use and productivity may be nuanced; employees’ views related to how using a sit-stand desk impacted their productivity were contingent on how they interacted with the desk within the organisational context. These findings are consistent with extant literature in which some sit-stand desk users have reported reduced concentration when standing compared to sitting, and others report that standing increases energy and alertness [17–19]. Given the strong influence of perceived productivity on sit-stand desk use, the findings indicate that workplaces should have a clear communication strategy alongside sit-stand desk provision, emphasising the evidence that standing to work does not reduce work efficiency. This may facilitate organisational cultural acceptance of sit-stand desks and address concern about colleagues’ perception of one’s behaviour and productivity. Controlled, experimental studies have found that taking short breaks from work activities can facilitate *increased* productivity (e.g. [44]). However, the present study and related literature [13] highlight that in practice in organisations, employees symbolise taking breaks as time-wasting, indicating that using a sit-stand desks is a more acceptable strategy for reducing workplace sitting than strategies that involve leaving the desk.

The present analysis highlights that decisions about whether to sit or stand at work are conditioned by social rules related to interactions between people. The shared meaning attached to individuals’ choice of postural and physical positioning in social (work) spaces can dissuade employees from using the sit-stand desk to stand when their colleagues are all sitting. Additionally, the act of sitting and standing is symbolised in terms of power differentials; being physically elevated represents authority over others [45]. Work organisations are inherently hierarchical and permeated with relations of power; sit-stand desk users with more senior positions may feel uneasy highlighting their seniority, and those with more junior positions may experience social discomfort as a result of contradicting the formal organisational power hierarchy by standing whilst more senior colleagues are sitting. A recent qualitative analysis of employees’ experience of standing in normally seated workplace meetings also highlighted how standing as a symbolisation of power affected how they felt about standing [46]. If the employee was leading the meeting, standing was voiced to be an appropriate method for denoting leadership, whereas if the employee was not leading the meeting employees often felt discomfort standing as they did not wish to be construed as exercising more power than they had within the meeting context [46]. Some participants felt guilty about having negative feelings about their sit-stand desk when positioned near colleagues with seated desks who expressed a desire to have a sit-stand desk. An immediate consequence of feeling guilty is that employees may feel compelled to stand more frequently or for longer. However, ultimately, such an emotional response is likely to reinforce negative feelings about sit-stand desks by employees. Social-cultural factors shape the acceptability of sit-stand desk use amongst employees; workplaces might consider the physical positioning of employees in the office space prior to implementing sit-stand desk or other sedentary behaviour reduction interventions.

The sit-stand desk intervention facilitated organisational cultural change towards a less sedentary working environment. Complex processes that incorporate various interacting factors underpin cultural change. Factors such as the increased national and international media attention on the health risks of prolonged workplace sitting [41], likely interacted with the delivery of the sit-stand desk intervention, to elicit top bottom-up and top down cultural change processes within the participating organisations. Thus, the sit-stand desk intervention *contributed* to (a) a process of normalisation of standing in the office-based workplace, and (b) an increased acceptance of sit-stand desks by on-the-ground employees and leaders. However, it is unlikely that the impact on sitting behaviour of employees not participating in the trial was widespread or substantial as most did not have unrestricted access to a sit-stand desk.

### Strengths and limitations

This study contributed to the advancement of approaches to examining the feasibility and acceptability of workplace interventions. Whilst many qualitative evaluations utilise interview methods, observation methods are rarely employed. Conducting observations involves data collection within the naturalistic setting and thus permits direct access to organisational processes and employee interactions [47], which was beneficial for examining organisational culture and contextualising interview data. Utilising these methods also permitted an examination of the impact of the intervention on the organisational setting [48], including organisational cultural change.

A limitation of the participant interview component of the study is that interviewees are predisposed to positive attitudes towards sit-stand desks, as they self-selected to partake in a workplace intervention that included sit-stand desk provision. Findings from a study of responses to workplace sitting reduction guidelines suggest that some people have intense negative feelings regarding the provision of sit-stand desks to office-based employees [42]. Future intervention studies could interview employees that actively chose not to participate in the intervention, to gain a more rounded view of the acceptability of sit-stand desks. Doing so would also permit a first-hand account of the impact of others’ sit-stand desk use on employees that do not have one, or do not wish to use one. For example, it would permit examination of sit-stand desk users’ suggestion that other employees may feel coerced into using a sit-stand desk against their will due to the social connotations connected to health behaviours, such as those that perform ‘healthy’ behaviours being more ‘disciplined’ than those that don’t.

## Conclusions

The present study investigated employees’ experiences of using sit-stand desks as part of a workplace intervention, within two UK office-based workplaces. Gaining an understanding of the feasibility and acceptability of using sit-stand desks is important for informing the development of guidance for workplaces looking to develop and implement workplace sedentary behaviour-reduction interventions, to improve their chance of success. A noteworthy strength of the present study is the *theoretical* analysis of intervention efficacy and acceptability, as a recent systematic review identified that only 36% of process evaluations of workplace health interventions utilised a theoretical framework [49]. This study evidences that organisational cultural theory and product design theory are relevant theories to underpin the evaluation of workplace sit-stand desk interventions, as they helped explain the mechanisms underpinning why and why not sit-stand desks were used and were viewed as acceptable by employees. The applicability of organisational cultural and product design theory to the data also provides empirical support to the theories. This study demonstrates that feasibility, acceptability and efficacy are not inherent within interventions, but rather a range of individual and contextual factors interact with the intervention to shape how employees (differently) experience the intervention, which indicates that sit-stand desks are not a one-size-fits-all solution for reducing sitting amongst office-based employees. The findings provide empirical support to the socio-ecological model as they indicate that a variety of complex and overlapping factors at the individual, environmental and organisational level shape employees’ response to using a sit-stand desk. Sit-stand desk interventions should be tailored to the organisational culture and context to improve their acceptability amongst employees.

## Supplementary information

**Additional file 1.** Example interview guide – for interviewees in the SS-O or SS-MC intervention arm.

## Data Availability

The dataset generated and analysed during the current study are not publicly available to reserve the anonymity of research participants but are available from the corresponding author on reasonable request.

## Abbreviations

MSD: Musculoskeletal discomfort
RCT: Randomised Controlled Trial
SS-MC: Multi-component sit-stand desk group
SS-O: Sit-stand desk only group
