# Supplementary material for: “Why would you want to stand?” an account of the lived experience of employees taking part in a workplace sit-stand desk intervention
Source: BMC Public Health. 2019 Dec 17;19:1692. doi: 10.1186/s12889-019-8038-9 (PMC6918567; doi:10.1186/s12889-019-8038-9)
Supplement: Supplementary file 1 — Additional file 1. Example interview guide – for interviewees in the SS-O or SS-MC intervention arm. [file 12889_2019_8038_MOESM1_ESM.docx]

Example interview guide – for interviewees in the SS-O or SS-MC intervention arm

| *Questions* | *Socio-ecological model* | *Organisational cultural theory* | *Product design theory* |
| --- | --- | --- | --- |
| 1. **Why did you initially apply to take part in the sit-stand project*?***   PROMPTS: Become more active, health problems  Work reasons, influence of colleagues / managers  Is being physically active important to you (social / physical benefits)  Are you active outside work? | Individual  Social / organisational  Social / individual  Community | -  Values  -  - | -  -  -  - |
| 1. **What are your views on being active at work?**   PROMPTS: Should you be/ have you been given the opportunity to be active at work?  Do you feel that [Workplace] values your activity and health at work?  Is there anything that makes it easier or harder to be active at work?  Is it appropriate to reduce sitting in the workplace?  Is your health at work a priority to you? | Organisational  Organisational  Organisational  Organisational/ societal  Societal /individual | Values / artefacts  Assumptions / values / legitimation environment  Artefacts  Assumptions  Assumptions | -  -  -  User / situation |
| 1. **What is your role at [workplace]?**   PROMPTS: Typical workday, desk work, meetings, travel  Does the type of work you do impact on your sitting, activity and health at work? | Organisational  Organisational | -  Artefacts | Situation  Situation |
| 1. **Did you have any expectations about your sit-stand desk?**   PROMPTS: the workstation itself, how much you would use it, any benefits or challenges  How did you select a desk? What were the reasons behind your choice? | Environmental  - | -  - | Aesthetic experience / usability / social design  Aesthetic experience / usability / social design |
| 1. **Were you there on the install day? If yes, what do you remember from this day?**   PROMPTS: Initial reaction to your desk? Did your feelings change over time?  Was the install disruptive to you/colleagues?  Were you happy with how it was installed? Did someone show you how to use it and was this helpful? Did you feel confident in knowing how to use it? | -  Social  Environmental | -  -  - | Aesthetic experience  Situation  Situation |
| 1. **What influences your use of yours sit-stand desk?**   PROMPTS: What prompts you to switch positions?  Any work-related, social, physical factors that make you use it more or less? | Environmental  Organisational/ social / individual | -  Assumptions / values | Usability /situation  Usability / person-product-situation interaction |
| 1. **Do other people at [workplace] influence how much you use your desk or what you think or feel about it?**   PROMPTS: pressure to use it  Are there any other people around you that have a sit-stand desk?  Disrupting others  Have you had an impact on others? | Social  Social  Social  Social  Social | -  Assumptions / values  -  Assumptions / values  Artefacts | Situation  Situation  -  Ease of use / situation  - |
| 1. **Has being part of the project influenced your views on how you think about your lifestyle in relation to activity, inactivity and health?**   PROMPTS: Influences on your activity in non-work contexts or non-desk-based work contexts  Importance of PA/health, awareness  Monitoring and diaries? | Community  Individual  Environmental / Individual | -  -  - | -  -  - |
| 1. **Do you have anything else that you wanted to say about the project, your experience of using the workstation, or activity, inactivity and work in general?** | - | - | - |

Example interview guide – for interviewees in the control arm

| *Questions* | *Socio-ecological model* | *Organisational cultural theory* |
| --- | --- | --- |
| 1. **Why did you initially apply to take part in the sit-stand project*?***   PROMPTS: Become more active, health problems  Work reasons, influence of colleagues / managers  Is being physically active important to you (social / physical benefits)  Are you active outside work? | Individual  Social / organisational  Social / individual  Community | -  Values  -  - |
| 1. **What are your views on being active at work?**   PROMPTS: Have you been given the opportunity to be active at work?  Do you feel that [Workplace] values your activity and health at work?  Is there anything that makes it easier or harder to be active at work?  Is it appropriate to reduce sitting in the workplace?  Is your health at work a priority to you? | Organisational  Organisational  Organisational  Organisational/ societal  Societal /individual | Values / artefacts  Assumptions / values / legitimation environment  Artefacts  Assumptions  Assumptions |
| 1. **What is your role at [workplace]?**   PROMPTS: Typical workday, desk work, meetings, travel  Does the type of work you do impact on your sitting / activity / health at work? | Organisational  Organisational | -  Artefacts |
| 1. **How did you feel when told you wouldn’t be getting a sit-stand desk?** | Environmental | - |
| 1. **Are there any other people with sit-stand desks around you at work?**   PROMPTS: How does this make you feel? Does it affect you in any way? Does it have any influence on how active you are at work or on your work team?  Productivity and professionalism – seen as more or less productive or advantageous to be seen standing at work or being active at work? | Social / environmental / individual  Individual / societal | -  Assumptions / values / artefacts |
| 1. **Has being part of the project influenced your views on how you think about your lifestyle in relation to activity, inactivity and health?**   PROMPTS: Activity in non-work contexts or non-desk-based work contexts  Importance of PA/health, awareness  Monitoring and diaries? | Community  Individual  Environmental / Individual | -  -  - |
| 1. **Do you have anything else that you wanted to say about the project, or activity, inactivity and work in general?** | - | - |
